# Assessing aetiological overlap between child and adult attention-deficit hyperactivity disorder symptoms in an extended family design

**DOI:** 10.1192/bjo.2023.554

**Published:** 2023-09-06

**Authors:** Daniel L. Wechsler, Fruhling V. Rijsdijk, Nicoletta Adamo, Espen M. Eilertsen, Yasmin I. Ahmadzadeh, Isabella Badini, Laurie J. Hannigan, Eivind Ystrom, Tom A. McAdams

**Affiliations:** Department of Psychology, Institute of Psychiatry, Psychology and Neuroscience, King's College London, UK; Social, Genetic and Developmental Psychiatry Centre, Institute of Psychiatry, Psychology and Neuroscience, King's College London, UK; and Department of Psychology, Faculty of Social Sciences, Anton de Kom University, Suriname; Social, Genetic and Developmental Psychiatry Centre, Institute of Psychiatry, Psychology and Neuroscience, King's College London, UK; and South London and Maudsley NHS Foundation Trust, London, UK; PROMENTA Research Centre, University of Oslo, Norway; and Centre for Fertility and Health, Norwegian Institute of Public Health, Oslo, Norway; Social, Genetic and Developmental Psychiatry Centre, Institute of Psychiatry, Psychology and Neuroscience, King's College London, UK; Department of Mental Disorders, Norwegian Institute of Public Health, Oslo, Norway; Nic Waals Institute, Lovisenberg Diaconal Hospital, Oslo, Norway; and Medical Research Council Integrative Epidemiology Unit, University of Bristol, UK; PROMENTA Research Centre, University of Oslo, Norway; Department of Mental Disorders, Norwegian Institute of Public Health, Oslo, Norway; and School of Pharmacy, University of Oslo, Norway; Social, Genetic and Developmental Psychiatry Centre, Institute of Psychiatry, Psychology and Neuroscience, King's College London, UK; and PROMENTA Research Centre, University of Oslo, Norway

**Keywords:** Comorbidity, aetiology, attention-deficit hyperactivity disorders, genetics, Medical Birth Registry of Norway

## Abstract

**Background:**

Several longitudinal studies have cast doubt on the aetiological overlap between child and adult attention-deficit hyperactivity disorder (ADHD). However, a lack of genetically sensitive data following children across adulthood precludes direct evaluation of aetiological overlap between child and adult ADHD.

**Aims:**

We circumvent the existing gap in longitudinal data by exploring genetic overlap between maternal (adult) and offspring (child) ADHD and comorbid symptoms in an extended family cohort.

**Method:**

Data were drawn from the Norwegian Mother, Father and Child Cohort Study, a Norwegian birth registry cohort of 114 500 children and their parents. Medical Birth Registry of Norway data were used to link extended families. Mothers self-reported their own ADHD symptoms when children were aged 3 years; reported children's ADHD symptoms at age 5 years; and children's ADHD, oppositional defiant disorder (ODD), conduct disorder, anxiety and depression symptoms at age 8 years. Genetic correlations were derived from Multiple-Children-of-Twins-and-Siblings and extended bivariate twin models.

**Results:**

Phenotypic correlations between adult ADHD symptoms and child ADHD, ODD, conduct disorder, anxiety and depression symptoms at age 8 years were underpinned by medium-to-large genetic correlations (child ADHD: *r_G_* = 0.55, 95% CI 0.43−0.93; ODD: *r_G_* = 0.80, 95% CI 0.46−1; conduct disorder: *r_G_* = 0.44, 95% CI 0.28−1; anxiety: *r_G_* = 0.72, 95% CI 0.48−1; depression: *r_G_* = 1, 95% CI 0.66−1). These cross-generational adult–child genetic correlations were of a comparable magnitude to equivalent child–child genetic correlations with ADHD symptoms at age 5 years.

**Conclusions:**

Our findings provide genetically sensitive evidence that ADHD symptoms in adulthood share a common genetic architecture with symptoms of ADHD and four comorbid disorders at age 8 years. These findings suggest that in the majority of cases, ADHD symptoms in adulthood are not aetiologically distinct from in childhood.

Attention-deficit hyperactivity disorder (ADHD) is a highly heritable neurodevelopmental disorder featuring impairing core symptoms of hyperactivity, impulsivity and inattention. The past decade has seen a shift toward widespread recognition that ADHD can affect adults as well as children, and a general consensus that most cases of childhood ADHD persist into at least young adulthood.^1^ Although most children with ADHD experience a reduction in their hyperactivity and impulsivity symptoms over time, inattention symptoms tend to remain relatively stable,^2^ and in a small but substantial portion of cases, one or more of these core symptoms have been shown to worsen over time.^3^ Despite these age-related differences, longitudinal twin studies have shown that ADHD symptoms in childhood, adolescence and early adulthood rely on partially overlapping genetic influences.^4,5^ However, studies of several longitudinal cohorts have reported that a majority of participants who met the diagnostic criteria for ADHD in adulthood did not meet the criteria in childhood, and *vice versa*.^6–8^ These findings represented a challenge to the notion of ADHD as an aetiologically consistent condition across the lifespan, renewing longstanding doubts as to whether putative cases of ADHD in adults should be as readily diagnosed and treated as undiagnosed or later-onset presentations of the neurodevelopmental disorder that has long been studied and treated in children.^9^

Since the initial studies suggesting the distinctiveness of childhood and adult ADHD first surfaced, various studies have sought to shed light on the topic.^10,11^ However, the evidence base on lifespan trajectories of ADHD remains quite limited. A recent review of longitudinal research by Asherson and Agnew-Blais^12^ found evidence for a late-onset group who met the diagnostic criteria for ADHD by early adulthood despite not having met the criteria in childhood.^12^ In many of these cases, symptoms occurred exclusively in the context of other disorders, broadly supporting suggestions that adult-onset ADHD symptoms could be better explained by other disorders or environmental factors, including substance use disorders.^13^ However, refuting the notion of overall aetiological distinction, Asherson and Agnew-Blais^12^ noted that many late-onset cases displayed subthreshold symptoms of ADHD as children, or met the criteria for common comorbid disorders, particularly oppositional defiant disorder (ODD). They also suggested that most late-onset cases could more accurately be classified as ‘adolescent-onset’, with symptoms first emerging at ages 12–16 years. Among their conclusions, they posited that there may be variation in age at onset for ADHD similar to that seen in other neurodevelopmental disorders, such as schizophrenia. This would suggest that most putative cases of ADHD in adults actually represent continuations (or otherwise later-onset presentations) of the neurodevelopmental disorder of ADHD seen in childhood.

## Addressing a gap in longitudinal data on ADHD symptoms across adulthood

Asherson and Agnew-Blais^12^ highlighted several important gaps in extant research, which preclude clear conclusions as to the overall continuity of ADHD symptoms from childhood to adulthood. Key among them was that most longitudinal childhood cohorts extend only to late adolescence or young adulthood, with a marked lack of data extending further into adulthood. Therefore, although it has been shown that most children with ADHD will still meet the diagnostic criteria by early adulthood, it remains unclear whether they will continue to do so across adulthood. Furthermore, the lack of genetically informative cohort data spanning this longer period prevents the direct assessment of aetiological overlap between ADHD symptoms in childhood and across adulthood. This gap in data is a key obstacle to drawing clear conclusions about whether ADHD in adulthood represents the same aetiological entity as childhood ADHD.

Recent genomic research has attempted to bridge this gap, providing some evidence for genetic overlap between ADHD in childhood and adulthood. Rovira et al^14^ reported a high correlation (*r_G_* = 0.81) between polygenic scores for ADHD ascertained in children (mean age 10.14 years, s.d. 3.24) and adults (mean age 33.46 years, s.d. 9.76). They also found significant correlations between child and adult ADHD polygenic scores and related phenotypes, including smoking, early pregnancy, academic performance and intelligence. An important caveat is that genomic findings continue to suffer from the missing heritability problem, explaining only a small portion of ADHD's total heritability compared with quantitative genetic estimates. For example, the genome-wide association study meta-analysis used by Rovira et al^14^ explained 17–19% of variance in ADHD symptoms in their child-only and combined child and adult samples, compared with estimates of 70–75% found in twin studies.^15^ Therefore, further research using methods capable of capturing all genetic variance in ADHD is also needed.

Very few studies currently exist that can directly address the above questions by following people from childhood to adulthood. One way to circumvent this problem is to use intergenerational family data to estimate genetic covariance between adult and child ADHD across generations. In the present article, we use this novel extended family approach to investigate the aetiological overlap between ADHD in childhood and adulthood, by assessing the extent to which adult ADHD symptoms in mothers are genetically related to symptoms of ADHD and several common comorbid disorders in their offspring. In doing so, we provide an indication of overall aetiological overlap between ADHD symptoms in adulthood and a broader pattern of ADHD-related symptoms in childhood. We do this using the Norwegian Mother, Father and Child Cohort Study (MoBa), a large data-set of related parents and their children.

## Method

### Sample

MoBa is a prospective, population-based birth registry cohort of 114 500 children, 95 200 birth mothers and 75 200 birth fathers in Norway.^16^ Data collection covered pregnancies across all of Norway from 1999 to 2008, with 40.6% of eligible pregnant women consenting to participate in the study. The current study is based on version 12 of the quality-assured data files released for research in January 2019. Using pedigree data from the Medical Birth Registry of Norway (MBRN), a national health registry containing information about all births in Norway, we used deterministic linkage via unique birth and parent numbers spanning several generations, to group MoBa participants into extended families of twins, siblings, half-siblings and cousins in both the parent and child generations. This analytic sample consisted of a total of 25 469 mothers who reported their own ADHD symptoms and/or at least one child measure for 30 833 of their children (51% male). Mothers were aged 17–45 years when children were born (mean age 29.97 years, s.d. 4.21). Supplementary Table 1 available at https://doi.org/10.1192/bjo.2023.554 displays frequencies of mothers and children stratified by maternal relatedness groups and child relatedness groups.

### Ethics

The establishment of MoBa and initial data collection was based on a license from the Norwegian Data Protection Agency and approval from The Regional Committees for Medical and Health Research Ethics. The MoBa cohort is currently regulated by the Norwegian Health Registry Act. The current study was approved by The Regional Committees for Medical and Health Research Ethics (approval codes PDB981/PDB2330).

### Measures

**Fig. 1:**
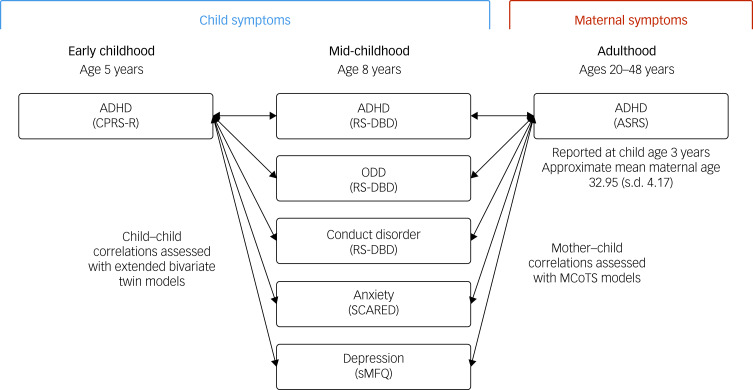
Overview of child and parent symptom measures used in the current study, wherein children's ADHD symptoms in early childhood (left) and maternal ADHD symptoms in adulthood (right) were assessed for aetiological overlap with children's ADHD, ODD, conduct disorder, anxiety and depression symptoms in mid-childhood (centre). ADHD, attention-deficit hyperactivity disorder; ASRS, Adult Self-Report Scale; CPRS-R, Conners’ Parent Rating Scale-Revised Short Form; MCoTS, Multiple-Children-of-Twins-and-Siblings; ODD, oppositional defiant disorder; RS-DBD, Parent/Teacher Rating Scale for Disruptive Behavior Disorders; SCARED, Screen for Child Anxiety Related Emotional Disorders; sMFQ, Short Moods and Feelings Questionnaire.

Figure 1 provides an overview of symptom measures used in the current study, wherein two ADHD measures (one of children's ADHD symptoms in early childhood and one of mothers’ ADHD symptoms in adulthood) were assessed for aetiological overlap with a set of five measures of children's ADHD and comorbid symptoms in mid-childhood. Internal consistency indices for all measures are displayed in Supplementary Table 2.

### Adult ADHD symptoms (reported by mothers when children were aged 3 years)

Mothers reported on their own ADHD symptoms by completing the Adult Self-Report Scale, a self-report screening tool for ADHD in adults which has high levels of concordance (area under the curve of 0.90) with clinical diagnoses.^17^ It includes six items measuring problems with sustained attention, task initiation and completion, organisation and age-appropriate indicators of hyperactivity (e.g. fidgeting when sitting still for prolonged periods). Mothers rated the frequency with which they experienced these problems in the past 6 months, on a five-point Likert scale of ‘never’, ‘rarely’, ‘sometimes’, ‘often’ and ‘very often’. Mothers were 20–48 years of age when reporting their ADHD symptoms (mean age of approximately 32.95 years, approximate s.d. 4.17).

### Child ADHD symptoms at age 5 years

Mothers reported children's ADHD symptoms at age 5 years by completing the Conners’ Parent Rating Scale-Revised Short Form, a well-validated measure of parent-reported child ADHD symptoms, including 12 items covering a range of hyperactive, impulsive and inattentive symptoms in children.^18^ Mothers rated the extent to which each item described their child's behaviour in the past month, on a four-point Likert scale of ‘not true’, ‘somewhat true’, ‘quite true’ and ‘very true’.

### Child ADHD and comorbid symptoms at age 8 years

Mothers reported children's ADHD, ODD and conduct disorder symptoms at age 8 years by completing the Parent/Teacher Rating Scale for Disruptive Behavior Disorders. This measure includes 18 items for ADHD symptoms, eight items for ODD symptoms and eight items for conduct disorder symptoms, based on DSM-IV criteria.^19^ Mothers rated the extent to which each item described their child's behaviour in the past year, on a four-point Likert scale of ‘never’, ‘rarely’, ‘sometimes’ and ‘often’. Mothers reported children's anxiety symptoms by completing the short version of the Screen for Child Anxiety Related Emotional Disorders, a measure covering symptoms of several DSM-IV anxiety disorders. The five-item short version has similar psychometric properties to the full 41-item scale.^20^ Mothers rated the extent to which each item described their child's recent behaviour, on a three-point Likert scale of ‘not true’, ‘sometimes true’ and ‘very true’. Finally, mothers reported children's depression symptoms by completing the Short Moods and Feelings Questionnaire, a 13-item measure of recent feelings, thoughts and behaviours related to low mood and based on the DSM-III-TR criteria of depression.^21^ Mothers rated the extent to which each item described their child's behaviour in the past 2 weeks, on a three-point Likert scale of ‘not true’, ‘sometimes true’ and ‘true’.

### Analyses

We set out to evaluate genetic overlap between adult (maternal) ADHD symptoms, and child (offspring) symptoms of ADHD and several comorbid disorders in mid-childhood. Mothers reported on their own ADHD symptoms several years before reporting on their children's symptoms of ADHD, ODD, conduct disorder, anxiety and depression (i.e. when children were aged 3 *v*. 8 years), reducing the risk of time-specific shared rater bias affecting our results. To contextualise our findings on adult ADHD, we also assessed associations between the above child symptoms at age 8 years and children's own earlier ADHD symptoms at age 5 years. Child ADHD symptoms at ages 5 and 8 years were assessed with distinct measures (see above).

Two sets of analyses were conducted, each using the OpenMx 2.18.1 package^22^ in R Statistics version 4.0.3 for Windows (https://cran.r-project.org/bin/windows/base/old/4.0.3). First, five Multiple-Children-of-Twins-and-Siblings (MCoTS) models assessed genetic relationships between mothers’ adult ADHD symptoms and child ADHD, ODD, conduct disorder, anxiety and depression symptoms at age 8 years. Second, five extended bivariate twin models assessed genetic relationships between children's early ADHD symptoms at age 5 years and their later ADHD, ODD, conduct disorder, anxiety and depression symptoms at age 8 years. We adapted these bivariate twin models to account for the additional degrees of genetic relatedness between siblings (50%), half-siblings (25%) and cousins (12.5%), and to constrain the shared environmental effect in cousins and paternal half-siblings to zero (as most cousins and paternal half-siblings do not share a household).

MCoTS analyses are an extension of the Children-of-Twins (CoT) design, a quasi-experimental method of determining the extent to which a parent–offspring association is attributable to shared genetic influences.^23^ Where bivariate twin models can estimate genetic influences on phenotypic covariance between twins, CoT models can estimate genetic influences on phenotypic covariance between parents and children. Using extended families of twin parents and children, these models derive their power from the comparison of parent–offspring correlations and avuncular correlations (those between children and their aunt/uncle). Namely, children of identical twins share the same proportion of genes with their parent as they do with their aunt or uncle, but share a rearing environment only with their own parent. This separation of genetic and environmental influences allows for inferences as to the extent to which covariance between parents and their offspring is explained by shared genetic influences, with any excess parent–offspring similarity suggesting direct phenotypic transmission through exposure to the parent phenotype. The MCoTS design extends CoT analyses to include sibling, half-sibling and cousin parents, and multiple children per parent. This allows for larger data-sets of extended families to be used, increasing statistical power. In previous work, we have demonstrated that MCoTS models are adequately statistically powered in the MoBa sample.^23^ The full MCoTS model specification is shown in Supplementary Fig. 3, and the extended bivariate twin model specification is shown in Supplementary Fig. 4.

To control for covariates, we regressed all symptom measures on maternal age, parity (mothers’ number of previous births) and children's year of birth, and all child symptom measures on child gender. The residual measures were then used in analyses.

## Results

Descriptive statistics of all measures are shown in Table 1. Model fit comparisons and standardised parameter estimates from preliminary univariate models testing the significance of genetic and shared environmental effects on adult ADHD symptoms are shown in Supplementary Tables 5a and 5b. Model fit comparisons from MCoTS and extended bivariate twin models, testing genetic effects on covariance between adult and early child ADHD symptoms and each child symptom measure at age 8 years, are shown in Supplementary Tables 6 and 7.

**Table 1:** Descriptive statistics of all raw measures (all mother-reported)

Measure	*n*	Mean	s.d.	Minimum	Maximum	Skew	Kurtosis	s.e.
Parity (number of previous births)	30 833	0.73	0.83	0	4	1.07	0.99	0.00
Maternal age at childbirth	30 833	30.12	4.18	17	45	0.03	−0.05	0.02
Year of childbirth	30 833	2005.68	1.78	2002	2009	−0.12	−0.84	0.01
Adult ADHD (ASRS)^a^	30 503	2.08	0.57	1	5	0.37	0.49	0.00
Child ADHD age 5 years (CPRS-R)	23 392	1.36	0.38	1	4	1.95	5.95	0.00
Child ADHD age 8 years (RS-DBD)	24 063	1.47	0.39	1	4	1.78	4.66	0.00
Child ODD age 8 years (RS-DBD)	24 021	1.43	0.39	1	4	1.38	3.11	0.00
Child conduct disorder age 8 years (RS-DBD)	24 010	1.10	0.19	1	3	2.92	11.42	0.00
Child anxiety age 8 years (SCARED)	24 027	1.20	0.24	1	3	1.66	4.11	0.00
Child depression age 8 years (sMFQ)	23 995	1.14	0.18	1	3	2.30	8.15	0.00

ADHD, attention-deficit hyperactivity disorder; ASRS, Adult Self-Report Scale; CPRS-R, Conners’ Parent Rating Scale-Revised Short Form; RS-DBD, Parent/Teacher Rating Scale for Disruptive Behavior Disorders; ODD, oppositional defiant disorder; SCARED, Screen for Child Anxiety Related Emotional Disorders; sMFQ, Short Moods and Feelings Questionnaire.

a. Includes 6435 mothers who provided self-ratings of ADHD symptoms, but did not provide ratings of child symptom ratings at the waves studied (these extra maternal ADHD data were included to improve heritability estimates of adult ADHD symptoms). All other descriptors include only those mothers who provided at least one child symptom rating.

**Fig. 2:**
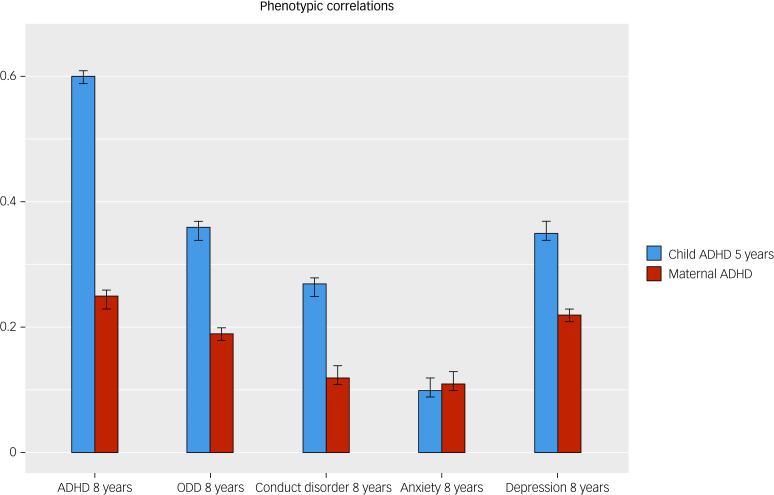
Bar plots displaying estimates (95% CI) of phenotypic correlations between each child symptom measure at age 8 years (on *x*-axis) and early child ADHD (blue bars) and adult ADHD (red bars). ADHD, attention-deficit hyperactivity disorder; ODD, oppositional defiant disorder.

**Fig. 3:**
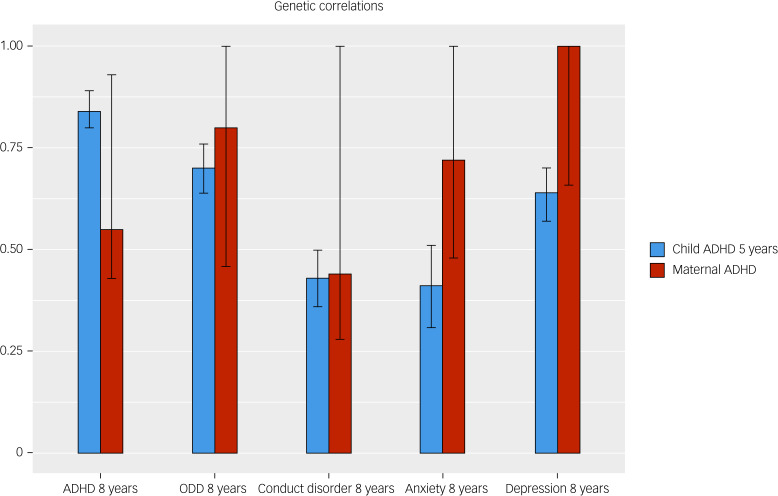
Bar plots displaying estimates (95% CI) of genetic correlations between each child symptom measure at age 8 years (on *x*-axis) and early child ADHD (blue bars) and adult ADHD (red bars). ADHD, attention-deficit hyperactivity disorder; ODD, oppositional defiant disorder.

Results from adult ADHD (MCoTS) and child ADHD (extended bivariate twin) models are shown in Figs 2 and 3. Phenotypic correlations followed an expected pattern, with child–child correlations being larger than intergenerational adult–child correlations. Genetic influences on adult ADHD symptoms were correlated with genetic influences on children's depression (*r_G_* = 1), ODD (*r_G_* = 0.80), anxiety (*r_G_* = 0.72), ADHD (*r_G_* = 0.55) and conduct disorder (*r_G_* = 0.44) symptoms at age 8 years. Similarly, genetic influences on child ADHD symptoms at age 5 years were correlated with genetic influences on child ADHD (*r_G_* = 0.84), ODD (*r_G_* = 0.70), depression (*r_G_* = 0.64), conduct disorder (*r_G_* = 0.43) and anxiety (*r_G_* = 0.41) symptoms at age 8 years. Standardised parameter estimates of all phenotypic and genetic correlations, including 95% confidence intervals, are shown in Supplementary Table 8.

Children's ADHD and comorbid symptoms at age 8 years shared roughly equal genetic overlap with adult ADHD symptoms and child ADHD symptoms at age 5 years (Fig. 3). Notably, for child ODD, conduct disorder, anxiety and depression symptoms, point estimates of genetic correlations with adult ADHD symptoms were higher than those of correlations with child ADHD symptoms at age 5 years. However, confidence intervals were markedly wider for adult ADHD estimates (with some upper bounds reaching 1), likely because of the lower power to estimate intergenerational genetic correlations in MCoTS models compared with twin genetic correlations in bivariate twin models.^23^ Importantly, none of the lower bounds of genetic correlations were close to zero, suggesting confidence in the genetic associations observed. Overall, confidence intervals of all adult ADHD genetic correlations were overlapping with their equivalent child ADHD correlations.

## Discussion

We set out to address a gap in research regarding the aetiological overlap between ADHD symptoms in childhood and adulthood. We did this by assessing the extent to which mothers’ adult ADHD symptoms and children's own early ADHD symptoms at age 5 years were genetically correlated with children's mid-childhood symptoms of ADHD and several common comorbid disorders at age 8 years. At the phenotypic level, children's own early ADHD symptoms were, unsurprisingly, more highly correlated with their later ADHD and comorbid symptoms by mid-childhood, as compared with mothers’ adult ADHD symptoms. However, genetic influences on both adult ADHD symptoms and early child ADHD symptoms shared similarly high correlations with genetic influences on children's ADHD and comorbid symptoms in mid-childhood.

Our findings provide novel evidence for a shared genetic architecture between ADHD symptoms in childhood and adulthood, insofar as the genetic influences on ADHD symptoms in mothers were highly correlated with genetic influences on ADHD symptoms in their children at age 8 years. Although past twin research has provided evidence for overlap between ADHD symptoms in childhood and late adolescence to early adulthood, no research to date has directly assessed genetic overlap between ADHD symptoms in childhood and across a broader age range in adulthood (i.e. beyond young adulthood).

More broadly, although there has been research on genetic overlap between ADHD and comorbid disorders in childhood,^24,25^ we are not aware of any research assessing the extent to which ADHD symptoms in adults share genetic overlap with children's symptoms of comorbid disorders. Our results demonstrate a very similar pattern of genetic overlap between ADHD symptoms measured at a range of ages in adulthood, and symptoms of ADHD and several common comorbid disorders in mid-childhood. This suggests that genetic influences on ADHD symptoms in adulthood substantially overlap with those on symptoms of ADHD and comorbid disorders in mid-childhood. In other words, it is unlikely that adult ADHD symptoms in our sample represent a distinct aetiological entity from child ADHD symptoms. Our results do not directly address nor rule out the possibility of a late-onset form of ADHD. However, if an aetiologically distinct later-onset form of ADHD exists in some adults, our findings suggest that it is quite rare in our sample, since it did not drastically affect our estimates of genetic overlap with children's ADHD and comorbid symptoms.

It is also notable that in a large sample of mothers and children not selected for clinically significant ADHD (most of whom would not meet diagnostic cut-offs), genetically driven correlations were found between mothers’ adult ADHD symptoms and children's ADHD and comorbid symptoms. This suggests that even if their symptoms are at subclinical levels, mothers transmit to their children genetic risk factors for the wider array of symptoms that co-occur with ADHD in clinically significant cases. In other words, mothers with, for example, mildly elevated hyperactivity or inattention may pass on genetic risk for mildly elevated impulsivity, oppositionality or anxiety in children. This is in line with growing evidence that psychiatric and neurodevelopmental disorders represent the extreme ends of normally distributed traits in the population, and co-occur as a rule, rather than being discrete and non-overlapping categories of severe disorder only present among those with specific risk factors.^26^

Although our study focused on ADHD and common comorbidities, future studies could apply our approach more broadly to investigate aetiological overlap between any pair of adult and child phenotypes, wherever cohorts of large extended families can be linked by twin, sibling and cousin relationships. An advantage of this method is that it controls for intervention effects at the within-person level, where relevant. For example, in conventional bivariate twin studies, if aetiological overlap were observed between children's ADHD symptoms and their later cardiovascular outcomes, this could, in part, be explained by the effects of ADHD medications on cardiovascular health. However, since most parents in established longitudinal cohorts to date have not received pharmacological treatment for ADHD, if our methodology were applied to investigate aetiological overlap between children's ADHD symptoms and their parents’ adult cardiovascular health outcomes, any overlap observed could not be explained by the potential effects of ADHD medications on cardiovascular health.

We investigated our hypotheses by using a large representative national birth registry cohort, including longitudinal child and parent data at a range of ages. Linking this data-set by extended family relationships with birth registry data allowed us to estimate genetic correlations between adult and child traits. This novel approach enabled us to bridge the gap in genetically sensitive data on ADHD and comorbid symptoms extending from childhood into adulthood. Another key strength of the study was that mothers reported their ADHD symptoms well into adulthood, with the youngest aged 20 years and the oldest aged 48 years at the time of self-report.

One limitation of our study was a lack of available data on ADHD symptoms in fathers, which could differ in their aetiological overlap with children's ADHD and comorbid symptoms. However, despite well-known gender differences in the prevalence and presentation of ADHD symptoms, genetic and environmental influences on ADHD have been shown not to differ significantly by gender.^27^ Nonetheless, future studies incorporating father-rated adult ADHD symptoms would be useful. Another potential limitation of our analyses was that mothers reported both their own and children's symptoms. Although maternal ratings of child symptoms are likely valid (as mothers typically spend the most time with their children), shared method bias could cause mothers’ child and self-ratings to be excessively correlated because of their own attitudes or traits.^28^ However, this is unlikely to have inflated our estimates of genetic overlap, as these rely on avuncular correlations, i.e. between an aunt's self-rated ADHD symptoms and their niece or nephew's mother-rated symptoms. Additionally, mothers reported their own ADHD symptoms and children's earlier and later symptoms several years apart (when children were aged 3, 5 and 8 years, respectively), reducing the likelihood of time-specific reporting biases. Systematic and/or heritable biases could still inflate estimates of genetic overlap; for instance, if both mothers in an extended family had higher ADHD symptoms, so tended to be less attentive to children's symptoms, and therefore consistently underreported these. However, several studies have reported a lack of evidence that maternal ADHD symptoms bias mothers’ reporting of their children's ADHD symptoms.^29,30^

In conclusion, our analyses address a lack of genetically sensitive research assessing the aetiological overlap between ADHD symptoms in childhood and throughout adulthood, as well as broader overlap with childhood comorbid symptoms. Our findings add to the evidence base for the continuity of childhood ADHD symptom into and across adulthood, by demonstrating a genetically driven co-occurrence of adult ADHD symptoms in mothers and a typical pattern of ADHD-related symptom in their children. Although these findings do not rule out the possibility of an aetiologically distinct form of adult ADHD in a minority of cases, they do provide genetically sensitive support for a common genetic architecture underpinning ADHD symptoms in a large, representative sample of children and adults.

Future studies could apply our approach to investigate aetiological overlap between a wider range of ADHD-related phenotypes in childhood and adulthood, or indeed, the overlap between any pair of adult and child phenotypes, where population registry data enable parents and children to be linked by extended family relationships into twin, sibling and cousin pairs. This constitutes a useful method of bridging the gap in genetically sensitive data for phenotypes when measures are available in childhood, but do not extend into adulthood.

## Supporting information

Wechsler et al. supplementary material Wechsler et al. supplementary material

## Data Availability

No new data were collected or analysed as part of the current study. Although access to MoBa and MBRN data is restricted, the MoBa data-set and its relevant overviews and data dictionaries are available at https://www.fhi.no/en/studies/moba/.

## References

[1] Kooij JJS, Bijlenga D, Salerno L, Jaeschke R, Bitter I, Balazs J, et al. Updated European Consensus Statement on diagnosis and treatment of adult ADHD. Eur Psychiatry 2019; 56: 14–34. 30453134 10.1016/j.eurpsy.2018.11.001

[2] Willcutt EG. The prevalence of DSM-IV attention-deficit/hyperactivity disorder: a meta-analytic review. Neurotherapeutics 2012; 9(3): 490–9. 22976615 10.1007/s13311-012-0135-8 PMC3441936

[3] Shaw P, Sudre G. Adolescent attention-deficit/hyperactivity disorder: understanding teenage symptom trajectories. Biol Psychiatry 2021; 89(2): 152–61. 32753233 10.1016/j.biopsych.2020.06.004 PMC7736482

[4] Chang Z, Lichtenstein P, Asherson PJ, Larsson H. Developmental twin study of attention problems: high heritabilities throughout development. JAMA Psychiatry 2013; 70(3): 311–8. 23303526 10.1001/jamapsychiatry.2013.287

[5] Kuntsi J, Rijsdijk F, Ronald A, Asherson P, Plomin R. Genetic influences on the stability of attention-deficit/hyperactivity disorder symptoms from early to middle childhood. Biol Psychiatry 2005; 57(6): 647–54. 15780852 10.1016/j.biopsych.2004.12.032

[6] Moffitt TE, Houts R, Asherson P, Belsky DW, Corcoran DL, Hammerle M, et al. Is adult ADHD a childhood-onset neurodevelopmental disorder? Evidence from a four-decade longitudinal cohort study. Am J Psychiatry 2015; 172(10): 967–77. 25998281 10.1176/appi.ajp.2015.14101266 PMC4591104

[7] Agnew-Blais JC, Polanczyk G, Danese A, Wertz J, Moffitt TE, Arseneault L. Persistence, remission and emergence of ADHD in young adulthood: results from a longitudinal, prospective population-based cohort. JAMA Psychiatry 2016; 73(7): 713. 27192174 10.1001/jamapsychiatry.2016.0465 PMC5475268

[8] Caye A, Rocha TB-M, Anselmi L, Murray J, Menezes AM, Barros FC, et al. Attention-deficit/hyperactivity disorder trajectories from childhood to young adulthood: evidence from a birth cohort supporting a late-onset syndrome. JAMA Psychiatry 2016; 73(7): 705–12. 27192050 10.1001/jamapsychiatry.2016.0383

[9] Castellanos FX. Is adult-onset ADHD a distinct entity? Am J Psychiatry 2015; 172(10): 929–31. 26423474 10.1176/appi.ajp.2015.15070988

[10] Faraone SV, Biederman J. Can attention-deficit/hyperactivity disorder onset occur in adulthood? JAMA Psychiatry 2016; 73(7): 655–6. 27191055 10.1001/jamapsychiatry.2016.0400

[11] Sibley MH, Rohde LA, Swanson JM, Hechtman LT, Molina BS, Mitchell JT, et al. Late-onset ADHD reconsidered with comprehensive repeated assessments between ages 10 and 25. Am J Psychiatry 2018; 175(2): 140–9. 29050505 10.1176/appi.ajp.2017.17030298 PMC5814300

[12] Asherson P, Agnew-Blais J. Annual research review: does late-onset attention-deficit/hyperactivity disorder exist? J Child Psychol Psychiatry 2019; 60(4): 333–52. 30843223 10.1111/jcpp.13020

[13] Taylor LE, Kaplan-Kahn EA, Lighthall RA, Antshel KM. Adult-onset ADHD: a critical analysis and alternative explanations. Child Psychiatry Hum Dev 2022; 53(4): 635–53. 33738692 10.1007/s10578-021-01159-w

[14] Rovira P, Demontis D, Sánchez-Mora C, Zayats T, Klein M, Mota NR, et al. Shared genetic background between children and adults with attention deficit/hyperactivity disorder. Neuropsychopharmacology 2020; 45(10): 1617–26. 32279069 10.1038/s41386-020-0664-5 PMC7419307

[15] Faraone SV, Larsson H. Genetics of attention deficit hyperactivity disorder. Mol Psychiatry 2019; 24(4): 562. 29892054 10.1038/s41380-018-0070-0 PMC6477889

[16] Magnus P, Birke C, Vejrup K, Haugan A, Alsaker E, Daltveit AK, et al. Cohort profile update: the Norwegian Mother and Child Cohort Study (MoBa). Int J Epidemiol 2016; 45(2): 382–8. 27063603 10.1093/ije/dyw029

[17] Kessler RC, Adler LA, Gruber MJ, Sarawate CA, Spencer T, Van Brunt DL. Validity of the World Health Organization Adult ADHD Self-Report Scale (ASRS) screener in a representative sample of health plan members. Int J Methods Psychiatr Res 2007; 16(2): 52–65. 17623385 10.1002/mpr.208 PMC2044504

[18] Conners CK, Sitarenios G, Parker JD, Epstein JN. The revised Conners’ Parent Rating Scale (CPRS-R): factor structure, reliability, and criterion validity. J Abnorm Child Psychol 1998; 26(4): 257–68. 9700518 10.1023/a:1022602400621

[19] Silva RR, Alpert M, Pouget E, Silva V, Trosper S, Reyes K, et al. A rating scale for disruptive behavior disorders, based on the DSM-IV item pool. Psychiatr Q 2005; 76(4): 327–39. 16217627 10.1007/s11126-005-4966-x

[20] Birmaher B, Brent DA, Chiappetta L, Bridge J, Monga S, Baugher M. Psychometric properties of the Screen for Child Anxiety Related Emotional Disorders (SCARED): a replication study. J Am Acad Child Adolesc Psychiatry 1999; 38(10): 1230–6. 10517055 10.1097/00004583-199910000-00011

[21] Messer SC, Angold A, Costello EJ, Loeber R, Van Kammen W, Stouthamer-Loeber M. Development of a short questionnaire for use in epidemiological studies of depression in children and adolescents: factor composition and structure across development. Int J Methods Psychiatr Res 1995; 5: 251–62.

[22] Neale MC, Hunter MD, Pritikin JN, Zahery M, Brick TR, Kirkpatrick RM, et al. OpenMx 2.0: extended structural equation and statistical modeling. Psychometrika 2016; 81(2): 535–49. 25622929 10.1007/s11336-014-9435-8 PMC4516707

[23] McAdams TA, Hannigan LJ, Eilertsen EM, Gjerde LC, Ystrom E, Rijsdijk FV. Revisiting the children-of-twins design: improving existing models for the exploration of intergenerational associations. Behav Genet 2018; 48(5): 397–412. 29961153 10.1007/s10519-018-9912-4 PMC6097723

[24] Tuvblad C, Zheng M, Raine A, Baker LA. A common genetic factor explains the covariation among ADHD ODD and CD symptoms in 9–10 year old boys and girls. J Abnorm Child Psychol 2009; 37(2): 153–67. 19015975 10.1007/s10802-008-9278-9 PMC2634815

[25] Michelini G, Eley TC, Gregory AM, McAdams TA. Aetiological overlap between anxiety and attention deficit hyperactivity symptom dimensions in adolescence. J Child Psychol Psychiatry 2015; 56(4): 423–31. 25195626 10.1111/jcpp.12318 PMC6607691

[26] Kotov R, Krueger RF, Watson D. A paradigm shift in psychiatric classification: the hierarchical taxonomy of psychopathology (HiTOP). World Psychiatry 2018; 17(1): 24. 29352543 10.1002/wps.20478 PMC5775140

[27] Larsson H, Chang Z, D'Onofrio BM, Lichtenstein P. The heritability of clinically diagnosed attention deficit hyperactivity disorder across the lifespan. Psychol Med 2014; 44(10): 2223–9. 24107258 10.1017/S0033291713002493 PMC4071160

[28] Deault LC. A systematic review of parenting in relation to the development of comorbidities and functional impairments in children with attention-deficit/hyperactivity disorder (ADHD). Child Psychiatry Hum Dev 2010; 41(2): 168–92. 19768532 10.1007/s10578-009-0159-4

[29] Faraone SV, Monuteaux MC, Biederman J, Cohan SL, Mick E. Does parental ADHD bias maternal reports of ADHD symptoms in children? J Consult Clin Psychol 2003; 71(1): 168. 12602437 10.1037//0022-006x.71.1.168

[30] Jassy JS. Maternal ADHD symptoms and maternal ratings of child ADHD symptoms: are more inattentive mothers less accurate? *Master's thesis* Department of Psychology, University of British Columbia, 2009.

