# Supplementary material for: Assessing aetiological overlap between child and adult attention-deficit hyperactivity disorder symptoms in an extended family design
Source: BJPsych Open. 2023 Sep 6;9(5):e169. doi: 10.1192/bjo.2023.554 (PMC10617499; doi:10.1192/bjo.2023.554)
Supplement: Wechsler et al. supplementary material [file S2056472423005549sup001.docx]

# Supplementary 1: Sample Frequencies

| Supplementary Table S1. Frequencies of mothers and children stratified by maternal relatedness and child relatedness, for paired extended families and for unpaired nuclear families (i.e. singleton mothers with more than one child in MoBa). | | |
| --- | --- | --- |
| **Extended families (N = 19,201)** |  |  |
| N stratified by the parent pairs used to identify extended families | **rA** | **N** |
| Identical twin pair | 1.00 | 60 |
| Full-sibling or fraternal twin pair | .500 | 12,085 |
| Maternal or paternal half-sibling pair | .250 | 690 |
| Cousin pair | .125 | 6,366 |
| N stratified by mothers’ relatedness in each extended family | **rA** | **N** |
| Identical twin pair | 1.00 | 43 |
| Full-sibling or fraternal twin pair | .500 | 4,074 |
| Maternal or paternal half-sibling pair | .250 | 261 |
| First cousin pair | .125 | 2,716 |
| Unrelated sisters/cousins-in-law pair | 0 | 12,107 |
| Number of offspring pairs linked to each mother | **rA** | **N** |
| Full-sibling pair | .500 | 5,089 |
| Maternal half-sibling pair | .250 | 41 |
| Unpaired (singleton) offspring | --- | 20,339 |
| **Unpaired nuclear families (N = 4,565)** |  |  |
| Number of offspring pairs linked to each mother | **rA** | **N** |
| Identical twin pair | 1.00 | 172 |
| Full-sibling or fraternal twin pair | .500 | 7,746 |
| Maternal half-sibling pair | .250 | 32 |

# Supplementary 2: Internal consistency

| Supplementary Table S2. Number of items and Cronbach’s alpha indicators of internal consistency for all raw measures (all mother-reported). | | |
| --- | --- | --- |
| **Measure** | **Number of items** | **Cronbach’s alpha (95% CI)** |
| Adult ADHD (ASRS) | 6 | .73 (.72, .73) |
| Child ADHD Age 5 (CPRS-R) | 12 | .88 (.87, .88) |
| Child ADHD Age 8 (RS-DBD) | 18 | .91 (.91, .91) |
| Child ODD Age 8 (RS-DBD) | 8 | .84 (.84, .84) |
| Child CD Age 8 (RS-DBD) | 8 | .70 (.70, .71) |
| Child Anxiety Age 8 (SCARED) | 5 | .47 (.47, .48) |
| Child Depression Age 8 (sMFQ) | 13 | .79 (.79, .79) |
| *ASRS = Adult Self-Report Scale; CPRS-R = Conners Parent Rating Scale-Revised Short Form; RS-DBD = Parent/Teacher Rating Scale for Disruptive Behavior; SCARED = Screen for Child Anxiety Related Disorders (SCARED); sMFQ = Short Moods and Feelings Questionnaire.* | | |

#
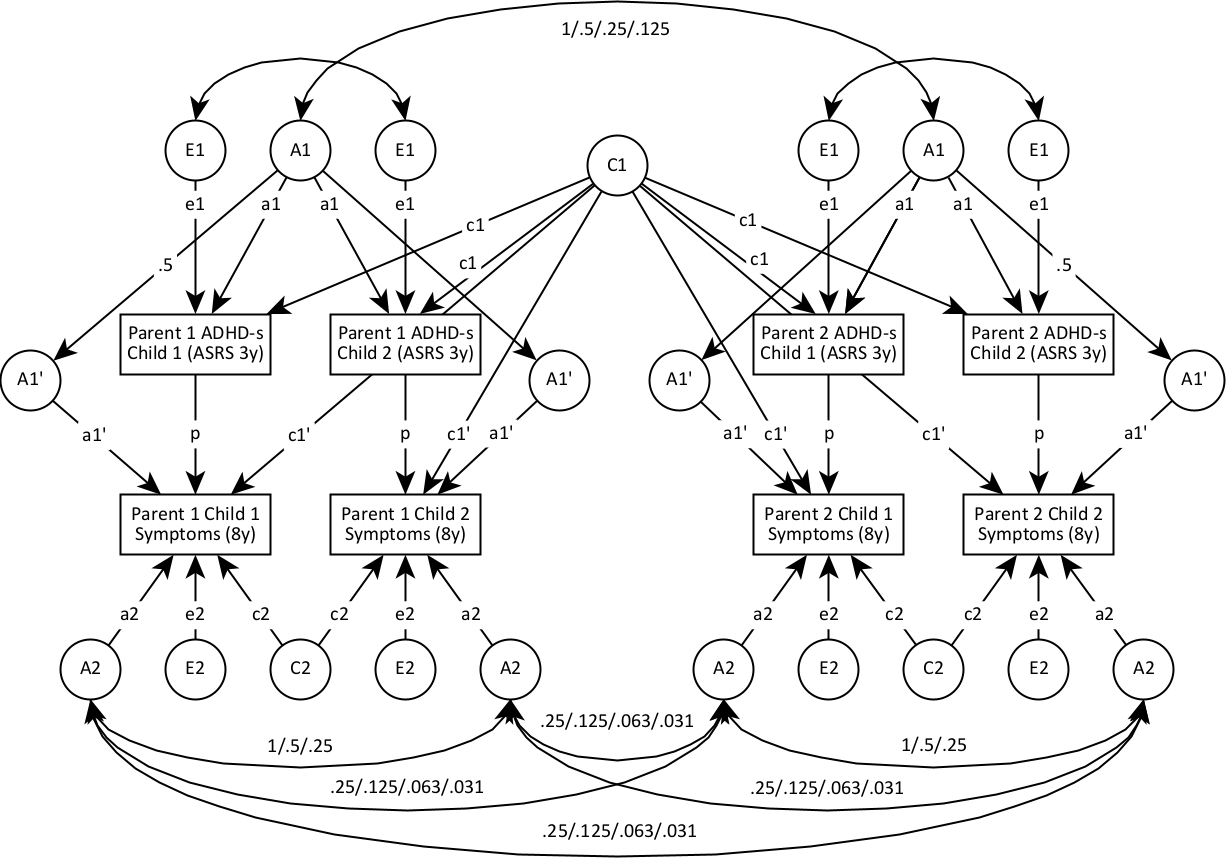
Supplementary 3: MCoTS Model Specification

**Supplementary Figure S3:** Model specification for the MCoTS model. A1, C1 and E1 latent factors represent parental additive genetic, shared environmental and non-shared environmental variance components. A2, C2 and E2 factors represent the equivalent variance components for children. A1’ latent factors represent genetic variance components that are shared between the parent and child phenotype. Paths a1’, c1’, and p represent the proportion of the parent-child association explained by genetic transmission, extended family environmental influence, and residual association indicating an influence of exposure to the parental phenotype. *ADHD-s = ADHD symptoms.*

#

# Supplementary 4: Extended Bivariate Twin Model Specification


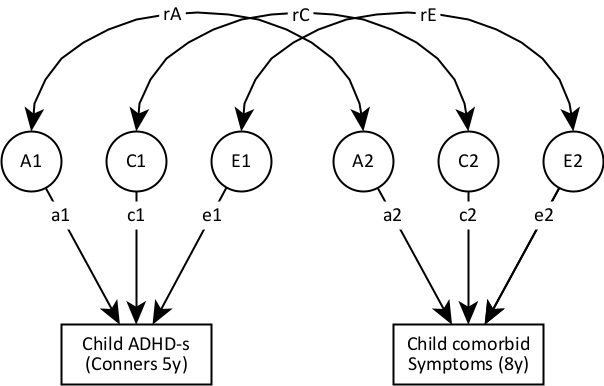


**Supplementary Figure S4:** Model specification for the Extended Bivariate Twin Model, adapted from conventional bivariate twin models to include additional degrees of genetic relatedness between siblings (50%), half-siblings (25%), and cousins (12.5%), and to constrain the shared environmental effect in cousins and paternal half-siblings to zero (as most cousins and paternal half-siblings do not share a household). A1, C1 and E1 latent factors represent parental additive genetic, shared environmental and non-shared environmental variance components for child ADHD symptoms at age 5. A2, C2 and E2 factors represent the equivalent variance components for child comorbid symptoms at age 8. Paths rA, rC, and rE represent genetic, shared environmental, and nonshared environmental associations underpinning the total phenotypic association between the two measures. *ADHD-s = ADHD symptoms.*

# Supplementary 5: Preliminary Univariate Analyses

Fit comparison tables and standardised parameter estimates from preliminary univariate models for adult (maternal) ADHD symptoms. Constraining shared environmental effects (C) to zero did not result in significantly worse model fit, whereas further constraining genetic effects (A) to zero did result in worse model fit. This indicated that variance on adult ADHD symptoms was explained by genetic and non-shared environmental effects, with no significant effect of shared environment. In the accepted AE models, the heritability of adult ADHD symptoms was estimated at 26%. Given the lack of shared environmental influences on adult ADHD symptoms, we opted not to estimate shared environmental influences (C1) nor extended family environmental influences (C1’) on adult ADHD symptoms in MCoTS models.

| **Supplementary Table S5a. Fit comparison tables of nested models testing the significance of genetic and shared environmental effects for adult ADHD symptoms.** | | | | | | |
| --- | --- | --- | --- | --- | --- | --- |
| **Model** | ***-2LL*** | ***df*** | ***AIC*** | ***∆LL*** | ***∆df*** | ***p*** |
| Full ACE | 66488.79 | 23951 | 18586.79 |  |  |  |
| AE vs. ACE | 66488.79 | 23952 | 18584.79 | -1.29e-08 | 1 | 1 |
| E vs. AE | 66488.79 | 23953 | 18606.58 | 23.79 | 1 | **< .001** |

| **Supplementary Table S5b. Standardised parameter estimates (95% CI) of genetic, shared, and non-shared environmental effects on adult ADHD symptoms.** | | | |
| --- | --- | --- | --- |
| **Model** | **A** | **C** | **E** |
| Full ACE | 0.26 (0.17, 0.36) | <0.01 (0.00, 0.17) | 0.74 (0.64, 0.91) |
| AE | 0.26 (0.17, 0.36) | -- | 0.74 (0.64, 0.83) |
| E | -- | -- | 1 (1, 1) |

# Supplementary 6: MCoTS Model Fit Comparisons

| **Supplementary Table S6. Fit comparison tables of nested models testing the significance of genetic transmission from adult (maternal) ADHD symptoms to each of the five child symptom measures at age 8.** | | | | | | |
| --- | --- | --- | --- | --- | --- | --- |
| **Adult ADHD → Child ADHD 8y** | ***-2LL*** | ***df*** | ***AIC*** | ***∆LL*** | ***∆df*** | ***p*** |
| Full model vs. | 150441.1 | 54444 | 41553.10 |  |  |  |
| No genetic transmission (A1’ = 0) | 150583.4 | 54445 | 41693.38 | 142.28 | 1 | **<.001** |
| **Adult ADHD → Child ODD 8y** |  |  |  |  |  |  |
| Full model vs.: | 150903.8 | 54433 | 42037.77 |  |  |  |
| No genetic transmission (A1’ = 0) | 151013.3 | 54434 | 42145.27 | 109.50 | 1 | **<.001** |
| **Adult ADHD → Child CD 8y** |  |  |  |  |  |  |
| Full model vs.: | 151563.2 | 54486 | 42591.23 |  |  |  |
| No genetic transmission (A1’ = 0) | 151609.4 | 54487 | 42635.45 | 46.22 | 1 | **<.001** |
| **Adult ADHD → Child Anxiety 8y** |  |  |  |  |  |  |
| Full model vs.: | 151737.7 | 54450 | 42837.69 |  |  |  |
| No genetic transmission (A1’ = 0) | 151799.0 | 54451 | 42897.03 | 61.34 | 1 | **<.001** |
| **Adult ADHD → Child Depression 8y** |  |  |  |  |  |  |
| Full model vs.: | 150736.0 | 54418 | 41900.05 |  |  |  |
| No genetic transmission (A1’ = 0) | 150871.2 | 54419 | 42033.18 | 135.13 | 1 | **<.001** |

# Supplementary 7: Extended Bivariate Twin Model Fit Comparisons

| **Supplementary Table S7. Fit comparison tables of nested models testing the significance of genetic and shared environmental overlap between child ADHD symptoms at age 5 and each of the five child symptom measures at age 8.** | | | | | | |
| --- | --- | --- | --- | --- | --- | --- |
| **Child ADHD 5y → Child ADHD 8y** | ***-2LL*** | ***df*** | ***AIC*** | ***∆LL*** | ***∆df*** | ***p*** |
| Full ACE | 126790.2 | 47370 | 32050.22 |  |  |  |
| AE vs. ACE | 126790.2 | 47373 | 32044.22 | -1.37e-08 | 3 | 1 |
| E vs. AE | 127500.9 | 47376 | 32748.93 | 710.71 | 3 | **<.001** |
| **Child ADHD 5y → Child ODD 8y** |  |  |  |  |  |  |
| Full ACE | 131527.0 | 47359 | 36809.04 |  |  |  |
| AE vs. ACE | 131527.5 | 47362 | 36803.50 | 0.46 | 3 | .93 |
| E vs. AE | 132322.6 | 47365 | 37592.65 | 795.15 | 3 | **<.001** |
| **Child ADHD 5y → Child CD 8y** |  |  |  |  |  |  |
| Full ACE | 132705.1 | 47412 | 37881.09 |  |  |  |
| AE vs. ACE | 132705.1 | 47415 | 37875.09 | -1.40e-08 | 3 | 1 |
| E vs. AE | 133408.0 | 47418 | 38572.05 | 702.95 | 3 | **<.001** |
| **Child ADHD 5y → Child Anxiety 8y** |  |  |  |  |  |  |
| Full ACE | 133814.8 | 47376 | 39062.79 |  |  |  |
| AE vs. ACE | 133814.8 | 47379 | 39056.79 | -7.74e-09 | 3 | 1 |
| E vs. AE | 134295.1 | 47382 | 39531.06 | 480.27 | 3 | **<.001** |
| **Child ADHD 5y → Child Depression 8y** |  |  |  |  |  |  |
| Full ACE | 131637.3 | 47344 | 36949.30 |  |  |  |
| AE vs. ACE | 131640.9 | 47347 | 36946.86 | 3.56 | 3 | .31 |
| E vs. AE | 132349.4 | 47350 | 37649.45 | 708.59 | 3 | **<.001** |

| **Supplementary Table S8. Standardised parameter estimates (95% CI) of phenotypic and genetic correlation coefficients between child ADHD symptoms at age 5, adult (maternal) ADHD symptoms, and each of the five child symptom measures at age 8.** | | | | | | |
| --- | --- | --- | --- | --- | --- | --- |
| **Child measure at 8y** |  | **Child ADHD 5y** | |  | **Adult ADHD** | |
|  |  | rPh | rA |  | rPh | rA |
| Child ADHD 8y |  | .60 (.59, .61) | .84 (.80, .89) |  | .25 (.23, .26) | .55 (.43, .93) |
| Child ODD 8y |  | .36 (.34, .37) | .70 (.64, .76) |  | .19 (.18, .20) | .80 (.46, 1) |
| Child CD 8y |  | .27 (.25, .28) | .43 (.36, .50) |  | .12 (.11, .14) | .44 (.28, 1) |
| Child Anxiety 8y |  | .10 (.09, .12) | .41 (.31, .51) |  | .11 (.10, .13) | .72 (.48, 1) |
| Child Depression 8y |  | .35 (.34, .37) | .64 (.57, .70) |  | .22 (.21, .23) | 1 (.66, 1) |

# Supplementary 8: MCoTS and Extended Bivariate Twin Model Parameter Estimates
